# News and Miscellany

**Published:** 1904-06

**Authors:** 


### News and Miscellany.

See Roster of State Medical Association of Texas in advertising pages.

---

When you write to my advertisers, mention the "Red Back," please. It is money in my pocket.

---

Dr. W. E. Fowler, physician to the penitentiary at Huntsville, was the first prison physician in the world, so far as I know, to segregate the consumptive convicts and take other precautions against this infection.

Will Repair Your Electrical Machines.—Static and all electrical medical apparatus put in running order. I am also agent for electrical and X-Ray apparatus. Oliver Brush, 710 Colorado Street, Austin, Texas.

---

Dr. Daniel's Paper on "The Cause and Prevention of Rape; Sadism in the Negro," read at the recent meeting of the State Medical Association of Texas, at Austin, can be had in pamphlet form for 10 cents. The Texas Medical Journal, Austin, Texas.

---

Dr. Robert Bartholow, of Philadelphia, died at his home May 10, 1904, aged 72 years. He was a distinguished author and teacher, first at Cincinnati, and then at Philadelphia, and had been an active and honorary members of numerous medical societies.

---

Special Reqeust.—If any of my readers received the May number without the pictures of the President of the State Medical Association, or the "Mascot," I will take it as an especial favor if they will let me know it; and I will, with pleasure, send the pictures. I have reason to believe that a few copies left the bindery minus the engravings.

---

Good Location Available.—For sale, a large fine residence, barn and stables, separate office near by. Seven acres of fine land. Price, $3000; half cash, balance to suit. Practice averages $1500 to $3000. Collections 98 per cent. Practice goes with property. Pretty village in a beautiful and rich farming section peopled with German and Bohemian settlers; prompt pay. Nearest competition ten miles. Dr. Breezes, care Texas Medical Journal.

---

The editor of the *Journal American Medical Association* says: "We have often referred to the shortsightedness of the practitioner who does not take, or who fails to read, at least one weekly scientific medical journal, national in character, that he may keep up with the current practice of his profession. At the same time, he should take his local publication and support it enthusiastically. Such a journal has a sphere of usefulness as a representative of the physicians and as a mouthpiece of the organized profession of the territory it covers." Subscribe for the Texas Medical Journal—"The Red Back."

